# Single-fiber probes for combined sensing and imaging in biological tissue: recent developments and prospects

**DOI:** 10.1364/BOE.517920

**Published:** 2024-03-15

**Authors:** Jiawen Li, Stephen C. Warren-Smith, Robert A. McLaughlin, Heike Ebendorff-Heidepriem

**Affiliations:** 1 School of Electrical and Mechanical Engineering, The University of Adelaide, South Australia, 5005, Australia; 2 Institute for Photonics and Advanced Sensing, The University of Adelaide, South Australia, 5005, Australia; 3 Future Industries Institute, The University of South Australia, Mawson Lakes, South Australia, 5095, Australia; 4 Faculty of Health and Medical Sciences, The University of Adelaide, South Australia, 5005, Australia; 5 School of Physics, Chemistry and Earth Sciences, The University of Adelaide, South Australia, 5005, Australia

## Abstract

Single-fiber-based sensing and imaging probes enable the co-located and simultaneous observation and measurement (i.e., ‘sense’ and ‘see’) of intricate biological processes within deep anatomical structures. This innovation opens new opportunities for investigating complex physiological phenomena and potentially allows more accurate diagnosis and monitoring of disease. This prospective review starts with presenting recent studies of single-fiber-based probes for concurrent and co-located fluorescence-based sensing and imaging. Notwithstanding the successful initial demonstration of integrated sensing and imaging within single-fiber-based miniaturized devices, the realization of these devices with enhanced sensing sensitivity and imaging resolution poses notable challenges. These challenges, in turn, present opportunities for future research, including the design and fabrication of complex lens systems and fiber architectures, the integration of novel materials and other sensing and imaging techniques.

## 1. Introduction

Fiber sensing and imaging are both areas of active research and development. Fiber sensing has been widely used in areas such as structural health and environmental monitoring [1], and biomedical applications [2]. Optical imaging has played a crucial role in the life sciences, from providing insight into the cellular basis of tissue [3] through to visualizing cellular function, such as in neuronal networks [4]. The integration of these complementary modalities—sensing and imaging—within a single fiber allows for measurement of a range of physical and biological parameters while providing anatomical/morphological information [5,6]. It addresses an unmet demand for *in vivo* study of complex biological processes, which can be heterogenous in space and time and thus require simultaneous, co-located sensing and imaging measurements.

In particular, fluorescence-intensity-based sensing is well-suited for integration with fiber imaging where the fiber guiding light for imaging can also be utilized for fluorescence excitation and emission signal detection [7]. The first approaches to combine fluorescence-based sensing with imaging in a single fiber relied on incorporation of fluorescent sensing molecules on the end of the imaging fiber via silanization [8]. A series of such combined fluorescence-based sensing and imaging fiber probes were subsequently developed for the measurements of pH, pO_2_ and l-glutamate [9–11]. However, these combined probes were built using multi-core imaging fiber and were limited to only acquiring superficial images of the sample. By contrast, incorporation of an alternative imaging modality, such as optical coherence tomography (OCT), can provide depth-resolved, tomographic imaging capabilities.

OCT is a widely used high-resolution biomedical imaging modality that detects back-scattered light with high sensitivity (70-120 dB) to reconstruct depth-resolved images of tissue [12]. It is apt to be combined with fiber sensing [7] but also poses the fundamental challenge that the sensing molecules and associated coating [13,14] at the end of the imaging fiber are required to have high transparency to the OCT light to avoid image distortions. For ***physical*** sensing, a dense coating with no porosity and hence high transparency can be used as the fluorescent sensing coating as it only needs to respond to the environment via a physical effect such as a change in temperature or magnetic field. Hence, the first example of a single-fiber-based combined fluorescence sensing and OCT imaging probe was based on a transparent glass coating with temperature sensitive fluorescence. Inspired by single-fiber-based fluorescence and OCT imaging [15], a single double-clad fiber (with an outer cladding diameter of 125 µm and the entire fiber outer diameter of 250 µm, i.e., the same as standard single mode fiber) was used to combine fluorescence excitation and collection with OCT imaging in the same fiber [7,13].

In contrast to ***physical*** sensing, where the analyte in the outer sensing environment can be measured through longer range physical field effects, ***chemical*** sensing, as required for most biomedical applications [6,16], relies on the analyte to diffuse from the outer environment to the sensing molecules. For fluorescence based chemical sensing which uses coatings at the end of solid fibers or along the length of the core surfaces of air/glass microstructured fibers, this requirement is fulfilled by the sensing molecules being attached to the outer surface of, or embedded in, the coating. This enables the chemical reaction between the sensing molecules and the analyte species, which results in fluorescence being generated. However, coatings were found to considerably reduce light transmission in microstructured fibers for which the side of the core was coated with a thin nano-scale layer of polyelectrolyte [17], silane [18] or polymethylmethacrylate (PMMA) [19]. This observation indicates that polymer-based coatings may induce significant light scattering in/on the coating and thus be detrimental to OCT imaging which is dependent on detection of the back-scattered signal from tissue.

One approach to circumvent this challenge is the pre-mixing of the analyte with the fluorescent sensing molecules (also referred to as fluorophore) instead of coating the fiber with sensing molecules. For example, Chen *et al.* presented an all-fiber needle probe with 710 µm outside diameter to achieve OCT imaging and pH measurement [20] via detection of fluorescence generated by a pH-sensitive fluorophore injected into the biological tissue. However, this method of pre-mixing the fluorophore with the analyte can be challenging or impractical for *in vivo* applications for two reasons. One is that a specific fluorophore needs to be developed that is able to be well distributed in the target tissue *in vivo* while also being biocompatible and not harmful to the tissue. The other reason is that the pre-mixing of a fluorophore may change the biological response, invalidating the measurements. These challenges have motivated the development of a fluorescent sensing coating that is sensitive to the target analyte, has a high transparency (i.e. low scattering) and can be readily applied to the end of an optical fiber that can be used for both the fluorescence excitation and collection and the OCT signal, similar to the approach used for the physical sensing with a glass coating [7].

**Fig. 1.**
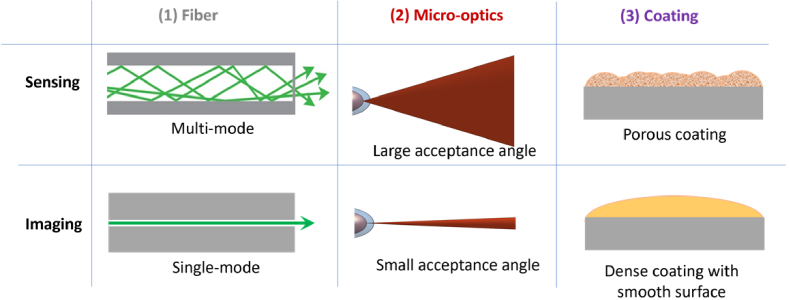
Schematics of the opposing property requirements for high performance sensing and imaging; **Fiber:** ray-optics model - multi-mode fiber enhances fluorescence signal collection for sensing whereas single-mode fiber enables high resolution for OCT imaging; **Micro-optics**: large acceptance angle enhances fluorescence signal collection for sensing whereas small acceptance angle provides large depth-of-focus for OCT imaging; **Surface coating**: porous coating enhances diffusion of analyte to sensing molecules within the coating whereas a high-transparency coating with smooth surface prevents image distortions.

The approaches used to date to integrate the two distinct modalities of sensing and imaging into a single-fiber-based probe have demonstrated the need for optimization of apparently opposing fiber, micro-optics, and coating property requirements for achieving high performance (Fig. 1).

• Fluorescence-based sensing benefits from ***multi***-mode and ***large*** acceptance angle fibers, and fluorescence-based chemical sensing usually takes advantages of porous coatings;
• OCT imaging benefits from ***single***-mode and ***small*** acceptance angle fibers, and high-transparency coatings with smooth surfaces.

In this prospective review paper, we first provide an overview of recent studies to address these apparently opposing requirements in the emerging field of developing combined fluorescence-based sensing and OCT imaging approaches (Section 2). We then present future directions for combining ***single***-mode and ***multi***-mode, achieving ***small*** and ***large*** acceptance angles, improving sensing coatings, applying 2D materials, and integrating other sensing/imaging techniques (Section 3). Finally, we discuss potential biomedical applications (Section 4).

## 2. Recent developments of single-fiber-based probes for combined sensing and imaging

### 2.1 Physical sensing combined with imaging

**Fig. 2.**
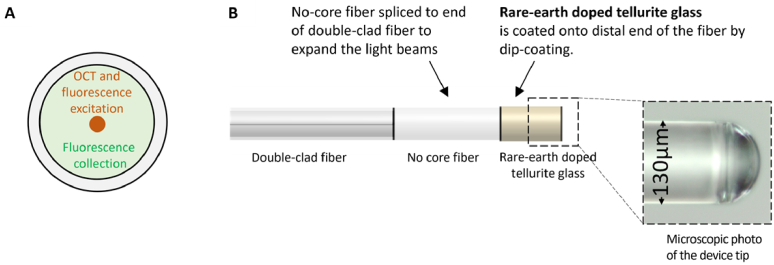
(A) Cross-sectional view of solid double-clad fiber indicating where OCT and fluorescence excitation (core: the orange dot) and fluorescence collection (inner cladding: the light green region) happened. (B) Schematic and microscopic photo of the temperature sensing + OCT imaging fiber probe.

One of the first examples of combining sensing and imaging modalities in a single fiber probe by using a fluorescent coating was demonstrated with a miniaturized temperature sensing + OCT imaging fiber probe (Fig. 2) for applications in deep tissue [7]. As illustrated in Fig. 2(A), the imaging function was generated via OCT using the core of a silica double-clad fiber, while the sensing function was achieved by sending fluorescence excitation light through the core of the fiber to the coating and collecting the majority of the fluorescence signal from the coating in the inner cladding of the fiber. The fluorescence-based temperature sensing was generated by ratiometric fluorescence measurement of a rare-earth ion doped tellurite glass i.e., a ‘ball-lens’ shaped coating at the distal end of the fiber. Specifically, the tellurite glass was co-doped with erbium ions, whose temperature-sensitive green fluorescence ratio enabled the temperature sensing function, and ytterbium ions, which enhanced the erbium green fluorescence and minimized autofluorescence through upconversion energy transfer [21]. The tellurite glass can accommodate high rare-earth doping concentration and has low phonon energy, which allows for efficient green fluorescence upconversion generation. Because the temperature at which tellurite glass becomes a liquid melt (∼700-800 °C) is lower than the temperature at which silica starts to deform (∼1200 °C), the tellurite glass could be coated onto the end of the silica fiber by dipping the silica fiber into the tellurite glass melt. This process created a half-sphere-shaped coating of tellurite glass (Fig. 2), which acted as a ball lens to focus the OCT imaging beam. In addition, because the tellurite glass has a higher refractive index of ∼2.0 compared to silica with index of ∼1.46, the tellurite glass coating generated a reflection at the fiber and tellurite interface, which was used as the reference reflection for a common-path OCT configuration [22]. The combined capability of temperature sensing and OCT imaging was proposed to characterize the effects of drug-induced hyperthermia.

This approach of using tellurite glass with fluorescence-based sensing function has potential to be extended to other sensing applications. For example, tellurite glass can be doped with nanodiamonds containing nitrogen vacancy color centers that are sensitive to magnetic field [23]. Coating of such a nanodiamond-doped tellurite glass onto the end of an OCT fiber may allow magnetic field sensing to be combined with OCT imaging.

### 2.2 Chemical sensing combined with imaging

In contrast to ***physical*** sensing, where a high-transparency, non-porous coating can be used, ***chemical*** sensing requires the coating to have sufficient permeability for the analyte to diffuse to the sensing molecules incorporated in the surface of or throughout the coating. For biomedical applications and integration with OCT, the coating is also required to be biocompatible and highly transparent. Early work used polyacrylamide coatings, which were demonstrated to be a viable coating for chemical sensing in biomedical settings [24,25] as this type of polymer could be readily functionalized with fluorophores and applied on fiber ends as a coating. However, the polyacrylamide coatings were found to induce high levels of noise in the OCT image (Supplement 1 Fig. S1).

**Fig. 3.**
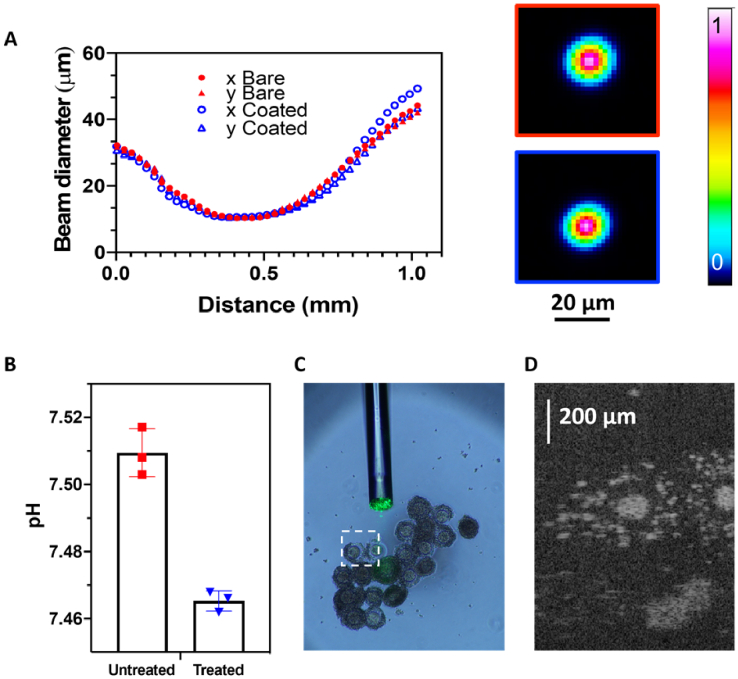
(A) Profiles of the OCT beam (with a central wavelength of 1310 nm) before and after silk coating, indicating transmission of light without distortion through the coating. (B-D) Sensing + imaging fiber probe used in IVF setting for pH measurement (B) Microscopic image of oocyte (C) OCT image of the oocyte by the sensing + imaging fiber probe. (D) Reprint from Reference 12 with permission.

Building upon initial work with silk fibroin functionalized with a fluorophore and applied as a coating onto a microstructured fiber [14], the group developed a silk-based coating method for pH sensing and imaging [13] (Fig. 3). To bind the pH sensitive fluorophore 5(6)carboxyseminaphthorhodafluor-2 (SNARF) to the silk fibroin matrix, the fluorophore was first covalently attached to a silk binding protein (SBP), which was then bonded to the silk fibroin protein by adding the SBP-SNARF conjugate to an aqueous solution of silk fibroin. By dipping the end of the optical fiber in this mixture, a fiber tip coating with embedded pH sensitive fluorophore was obtained. This silk + SBP-based coating demonstrates a unique combination of properties: it has sufficient transparency for the transmission of an OCT imaging beam with negligible beam distortion (Fig. 3(A)); it is stable to repeated washes of the optical fiber; and allows sufficient diffusion of the hydrogen ions as analyte for pH sensing. The performance of this fiber probe was demonstrated in an *in vitro* fertilization (IVF) setting to perform concurrent pH sensing and OCT imaging of oocytes (Fig. 3(B)-(D)). The results have potential to improve patient outcomes during IVF, by limiting the number of invasive follicle punctures required to collect sufficient oocytes.

It is important to note that pH sensing detects the concentration of H^+^ ions, which are the smallest ions and hence can easily diffuse through a coating. For the larger ions Al^3+^ and Ca^2+^ with different ionic radius, investigation of the performance of a fluorophore sensitive to both metal ions revealed that, when the fluorophore was embedded in a polymer coating, the larger Ca^2+^ ions showed lower fluorescence signal compared to the smaller Al^3+^ ions, demonstrating that the ability of the metal ions to diffuse through the coating to reach the fluorophore molecules is critical for sensing [26]. This hindrance of the diffusion of large size analyte species through a coating with embedded fluorophore hampers the sensing of large molecules such as proteins. To overcome this challenge, a new silk-based 2-step fiber coating method was developed, and its sensing performance was demonstrated using the biotin-streptavidin sensing model [27]. First, the fiber tip was coated with silk fibroin only, then the outer surface of the coating was decorated with SBP covalently bonded to biotin. For proof-of-concept, fluorophore tagged streptavidin protein was used as the analyte solution. This coating method with the SBP-biotin sensing component located at the outer coating surface showed significant fluorescence signal (even after washing the fiber tip), whereas the coating method with the SBP-biotin sensing component embedded in the silk fibroin coating did not show a fluorescence signal above the background noise. This result emphasizes that the precise location of the sensing molecules with respect to the fiber coating is a critical design consideration for optical fiber probes for sensing large molecules. The examples of silk-based coatings utilizing SBP-SNARF or SBP-biotin as the sensing component demonstrate the versatility of the silk-based coating platform to be tailored to a specific analyte by attaching the specific sensing molecule (e.g. fluorophore) to SBP, which is then bonded to the silk fibroin either inside the coating for sensing small species as for pH sensing, or on the outer surface of the coating for sensing large molecules such as proteins.

## 3. Future directions

Despite these promising preliminary demonstrations of combined sensing and imaging in single-fiber-based miniaturized probes, significant challenges remain in developing new and/or improved functionalities. Sensing and imaging have contrary requirements with some examples illustrated in Fig. 1. Combined multimodal fiber probes typically have poor optical performance for at least one modality [15,28], which limits their values in real-world applications. In this section, several directions are proposed to address these challenges by leveraging recent advances in other fields.

### 3.1 Optimized lens design for both sensing and imaging modalities

**Fig. 4.**
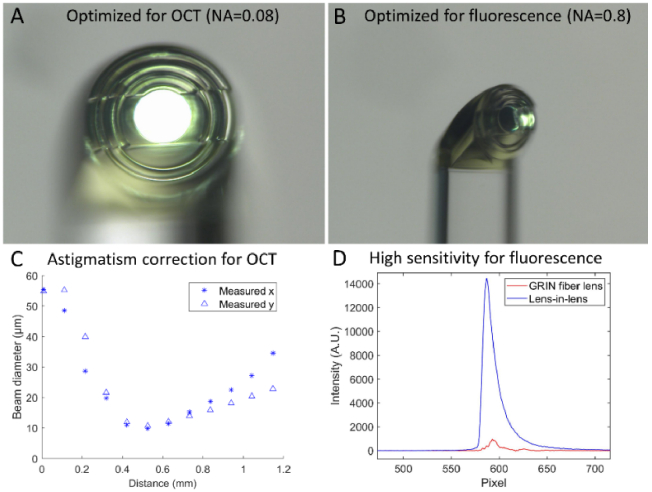
Design and characterization of a 3D-printed lens-in-lens endoscopic probe: Microscopic images of the 3D printed lens, highlighting the lens optimized for OCT (A) and the off-axis one for large NA fluorescence collection (B). (C) Beam profiles obtained from the 3D printed micro lens-in-lens design, demonstrating astigmatism correction for the OCT channel; (D) Comparison of collected indocyanine green fluorescence signals by a 3D printed lens-in-lens endoscope and GRIN-fiber-based design. Reprinted from Ref. [29] with permission under the Creative Commons CC-BY-NC license.

Various sensing and imaging modalities often require distinct optical lens designs to attain optimal measurements. Fluorescence-based modalities typically require a large numerical aperture (NA) to maximize capture of the fluorescence emission signal. Conversely, depth-resolved imaging with OCT requires low NA focusing optics to maximize the distance over which back-scattered light may by efficiently collected. However, merging these optical designs within a highly miniaturized single fiber device has been challenging. Recently, micro 3D printing, for example, two-photon polymerization 3D printing, can create complex shape directly on the tip of a fiber with fast and reliable transfer from design to production. This capability enables us to optimize lens design for multiple modalities. In particular, a novel sub-millimeter freeform lens-in-lens design that provides distinct but connected optical surfaces optimized for each modality was demonstrated, as shown in Fig. 4 [29]. The novel design comprises (i) an inner lens section with low NA of 0.08 and astigmatism correction for the OCT light beam to achieve a large depth of focus (Fig. 4(C)) and (ii) an outer lens section with high NA of 0.8 for the fluorescence light beam to achieve high collection efficiency (Fig. 4(D)). A similar freeform lens design is proposed for combining fluorescence-based sensing and OCT imaging to improve both the sensitivity of the sensing beam and the depth of focus of the imaging beam.

### 3.2 Utilization of specialty microstructured optical fibers

Microstructured optical fibers (MOFs), with their uniquely tailorable optical properties, provide a promising alternative for combining sensing and imaging. The suspended core MOF type has been demonstrated to be highly effective for fluorescence-based sensing [30–32] and for multi-core-based imaging [33–35] but there has been no demonstration of a MOF showing both modalities in a single fiber.

Compared to the traditional fiber tip sensing technique using solid-clad multi-mode fibers [36], the suspended core MOF type with small core and large air holes has been demonstrated to achieve a more than 50-fold improved sensitivity due to two effects. Firstly, this MOF type provides high acceptance angle [30] enhancing fluorescence signal collection. Secondly, it extends the interaction between the light and analyte along the fiber length by filling the internal holes with the analyte [37], greatly improving the fluorescence signal intensity. By utilizing fluorescence-based indicator molecules, suspended core MOFs have been used to sense chemical targets such as aluminum [37], zinc [38], and calcium [39] ions.

**Fig. 5.**
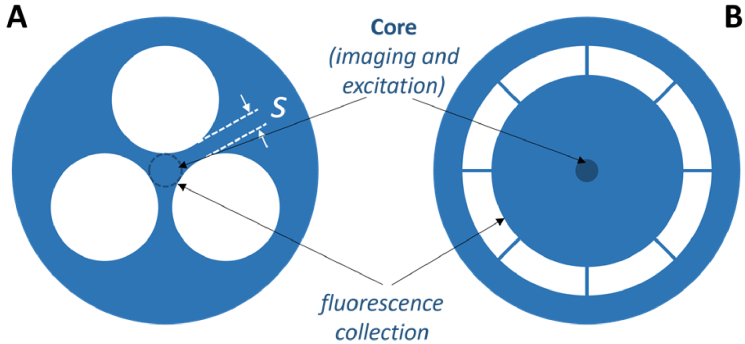
MOFs designed for adding (A) length integrated sensing, or (B) tip sensing to OCT imaging functionality.

The integration of the OCT modality requires overcoming the challenge that the suspended core MOF type typically supports multiple modes of the guided light. This is incompatible with OCT, which requires single mode guidance fibers. To take advantage of the high sensitivity of MOFs while supporting single-mode OCT imaging, two potential MOF designs are proposed (Fig. 5).

#### 3.2.1 Single mode MOF for length integrated sensing

Leaky mode MOF technology [40] allows achieving single-mode guidance in suspended core MOFs. This was shown for a MOF with inscribed fiber Braggs gratings for temperature sensing, where the thickness of the struts suspending the core (Fig. 5(A)) was optimized such that the multi-mode fiber became effectively single-mode at the wavelength of interest [36]. Such a fiber retains the ability of the large holes to be easily filled with an analyte for length integrated sensing. Another configuration that could be considered is the exposed core MOF type, which can also be used for length integrated fluorescence sensing [38], but with near instantaneous filling [41]. However, challenges remain in realizing a single mode core in the exposed core fiber.

#### 3.2.2 Double clad MOF for tip sensing

An alternative design is the double clad MOF (Fig. 5(B)), which has been used for fiber lasers [42–45]. This MOF type would allow for single mode OCT imaging in the central core and fluorescence collection in the inner cladding. This double clad fiber concept is similar to that of the sensing + imaging fiber probe described in [7], whereby the outer cladding was made of solid silica-based glass providing a low NA of 0.2 (see Fig. 2(A)). In contrast, the proposed double clad MOF design exhibits an outer cladding made of air holes (Fig. 5(B)), resulting in a large NA close to 1. This large NA together with the large size of the inner cladding would enable high efficiency in capturing fluorescence generated at the fiber tip.

### 3.3 Minimizing the impact of scattering from a coating

Coatings can reduce fiber transmission or generate image distortions, as reported previously [17–19,41,46]. This is likely to be caused by scattering either from inhomogeneities in the coating, coating thickness fluctuations and/or coating surface roughness. Further study is needed to better understand the causes for the scattering and ways to overcome it to improve image quality.

One approach to mitigate the impact of scattering is the spatial confinement of the coating to the sensing region. For example, in [7], the fluorescence excitation light was restricted to the central core. Alternatively, the excitation light can be coupled into the fiber such that most of it is guided in an inner cladding (in a fiber with either solid or air-hole outer cladding), which would enable spatial separation into a sensing region (the inner cladding) and an imaging region (the central core). In this case, protecting the core from being coated, possibly by applying a mask or by subsequent ablation and selective removal of the coating, would keep the imaging core free of a coating that induces deleterious scattering.

### 3.4 Photoswitchable coatings

The interaction between a fluorophore and its target analyte can be classified into irreversible and reversible. High-affinity interactions such as between strands of complementary oligomers, between antigen and antibody, or between metal ions and ligands are irreversible and, thus, can be only used for one-off measurement [47]. In contrast, interactions with sufficiently fast kinetics in both forward and reverse reactions allow the fluorophore to be reversible [48] and, thus, can be re-used and/or deployed for continuous measurements. For dynamic in vivo applications, photoswitchable fluorophores [49] are well suited for such applications as light is used both for switching-off, while the probe is first inserted and not at the right sensing location, and for switching-on, when the imaging probe has identified the right location for sensing. Thus, photoswitchable fluorophores are of particular interest for sensing + imaging fiber probes. Photoswitchable metal-ion-sensitive fluorophores have been integrated with fibers via two methods: (i) attached to the fiber end of traditional solid fibers or the core surface along the length of suspended core MOFs [50] or (ii) embedded in a coating on the core surface along the length of exposed core MOFs [38]. Both methods have potential to be used for coating an imaging fiber to achieve a re-usable sensing + imaging fiber probe.

### 3.5 Utilization of 2D materials

Novel materials, such as 2D materials [51,52] that have high surface area-to-volume ratios and adaptable optical properties, hold promise to enhance the sensitivity and imaging performance of single-fiber-based sensing + imaging probes. For example, graphene oxide (GO) and transition metal dichalcogenide were utilized for fluorescence-based sensing of metal ions such as Pb^2+^, Ag^+^, and Hg^2+^ [53–55]. GO has also been used to detect high-toxicity aflatoxin, where GO provides a high surface area to enhance the attachment of aflatoxin antibodies [56]. With excellent quenching properties to achieve controllable fluorescence turn-on process and/or by using the fluorescence resonance energy transfer from quantum dots, GO was also used for (bio)molecular sensing to detect DNA and different proteins [46,57]. Physical sensing, such as pressure sensing [58] and temperature sensing [59], was also realized. Traditional solid fibers were coated with 2D material at their fiber tips via various techniques including transferring 2D material films [60,61] and creating 2D-materials-polymer composites [62]. An important consideration in using 2D materials is to design the 2D material such that it causes negligible change to the beam at the imaging wavelength range, while providing sufficient sensitivity at the sensing wavelengths. A potential candidate is GO film, which was used as ultrathin lens for imaging via a fiber [60]. With these recent advances [46,53–55,57–60,62], it holds great hope to use 2D materials for combined sensing and imaging.

### 3.6 Integration of other imaging/sensing techniques

The integration of other more advanced imaging or sensing techniques, beyond fluorescence-intensity-based sensing and OCT imaging used in current sensing + imaging fiber probes, could further improve the capability of simultaneous sensing and imaging deep within living organisms. Examples of other techniques are multimode fiber imaging [63,64], fluorescence lifetime based imaging [65], photoacoustic tomography/sensing [66–68], and Raman scattering based sensing [69], especially stimulated or surface enhanced Raman [70], which can provide highly specific information about biological tissues and materials. There has been a continued interest in multimodal imaging via a single fiber, which has been discussed in various previous review papers [71,72]. In addition to multimodal imaging, multiplexed sensing of different species/parameters has also been an active area of research [6,73].

## 4. Potential biomedical applications

Single-fiber sensing and imaging probes provide quantitative, multidimensional, co-registered structural and molecular/physical data. These probes will not only measure a wide range of physiological parameters (sensing) to complement structural information (imaging), but also enable precise image-guided sensing through a single optical fiber. In this section, we give examples of possible real-world applications.

### 4.1 Precise image-guided sensing

Metal ions play critical roles in controlling cell survival, growth, and differentiation. In addition, some disorders and diseases, such as pathologic pain [74] or diabetes [75], are associated with abnormal concentrations of these ions. It is therefore essential to study the localized concentrations of these ions. However, they are often highly heterogeneous both in space and time and so monitoring their concentrations in local microenvironments remains challenging. Existing sensing techniques are highly sensitive and spatially localized [38,49], hence any misplacement of the probe can induce significant errors. Combined sensing + imaging fiber probes can address this limitation by using the real-time imaging function of the probe to precisely guide the placement of the co-localized sensing element. Once accurate placement is achieved, the sensing function of the probe could be utilized, enabling accurate measurement of metal ions at the target location. An example relevant to understanding chronic pain is measurement of the concentration of Zn^2+^ in the spinal fluid at a single lumbar region across different time points. Monitoring local Zn^2+^ sparks over time is of value for in vivo study to understand the mechanism of pain [76], ultimately allowing the evaluation of pain treatment options in the future.

### 4.2 Clinical applications

Single-fiber-based probes may help enable minimally invasive techniques to assess internal organs, guide biopsies [77] and other interventions [7]. For example, a temperature sensing + OCT imaging probe [7] has application in assessing the extent of tissue ablation in the treatment of liver cancer. Radiofrequency (RF) ablation is a common intervention to eliminate unresectable liver tumors [78]. For temperature-based therapies, such as RF ablation, cell death is closely related to accumulated thermal dose [79], which is dependent on both temperature increase and time of exposure. Currently, accurate RF ablation is difficult near blood vessels, as these impact the distribution to heat, and hence affect the region of tissue that will be ablated [80]. Although the dose of RF ablation can be estimated using theoretical models, it is impractical to comprehensively take the heat-sink effect of all blood vessels into consideration. This can result in incomplete ablation, and potential increases in local tumor recurrence rates. The combined temperature sensing and imaging fiber probe in [7] may detect blood vessels with OCT [81], while simultaneously measuring the real-time temperature changes. By optimizing RF dose according to the real-time measured temperature rises, it may be possible to more accurately control the tissue volume being ablated and treated.

Furthermore, single-fiber-based probes may also enable sensing + imaging of pathological features [82] or cancerous tissues [83], providing valuable information about tumor margins and tissue composition. They also have potential uses in cardiovascular imaging to visualize blood vessels, assess blood flow, and detect plaque buildup. Potential combined modalities are pH or nitric oxide sensing [84] combined with OCT imaging for plaque detection; or pressure sensing [85] with imaging for plaque detection and blood flow analysis.

Finally, yet importantly, biocompatibility of the single-fiber sensing + imaging probe needs to be considered for clinical applications. A summary about optical fiber and waveguide biocompatibility is provided in [86]. For the incorporation of chemical sensors in single-fiber probes, we note that silk is biocompatible and provides a stable coating material to attach sensors to fiber [87]. In addition, there are a range of novel coating materials available that can enhance the biocompatibility of the probe while providing an ‘antibacterial’ function [88,89]. Given the fragility of optical fibers, it is usually necessary to protect sensing + imaging probes in biocompatible casings prior to using them for in vivo scenarios. For endoscopic and intravascular applications, this will typically involve encasing the probe within a transparent, catheter sheath [29]. For applications involving insertion into solid tissue, it is also necessary to enhance the mechanical strength of the probe, typically using a rigid, often stainless steel, needle [7,28]. Such packaging also reduces the risk of cross-contamination between probe and tissue.

## 5. Summary and conclusions

Optical imaging enables in vivo 3D imaging of biological microstructures. The addition of fluorescence-based sensing within the same fiber would provide co-located molecular and microstructural information, giving a holistic view of complex biological activities. Such a probe has clinical application in situations where repeated, long-term measurements at exactly the same in vivo location are advantageous, for example the monitoring of local Zn^2+^sparks in a spinal cord, which would enable key fundamental biological questions to be answered and lead to better understanding and management of pain.

In the past decade, *physical* sensing with imaging and *chemical* sensing with imaging have been both demonstrated in various biomedical applications and can potentially lead to more clinical applications. As summarized in Table 1, the single-fiber-based sensing + imaging approach offers advantages such as compactness, flexibility, and the ability to perform multiple functions simultaneously. The utilization of MOF designs, photoswitchable fluorophores, 2D materials, and integration of other sensing and/or imaging technologies hold promise to further advance this technology and its real-world applications.

**Table 1.** Summary of single fiber imaging and sensing

	Main idea	Examples/Benefits
Why	Size	Being easy and safe to be inserted into the brain, blood vessel, small airway, and ducts, etc.
	Co-localization	Enabling meaningful measurement of a biological process which is highly heterogenous in space and/or time
When not to	Low benefit-cost ratio	When measuring in a homogenous and stable environment and/or in a large lumen where separate fibers for sensing and imaging can be easily placed inside
How (‘existing solutions’)	By using a multi-core imaging fiber and silanization	Fluorescent sensing molecules can be incorporated onto the end of the multi-core imaging fiber via silanization. The approach enables imaging of the surface, and the diameter of the fiber is usually larger than 250 µm depending on the number of fiber cores
	By using a double clad fiber and coating	A single double-clad fiber (with an outer cladding diameter of 125 µm) can be used to combine fluorescence excitation and collection with optical coherence tomography (OCT) imaging in the same fiber. This approach enables imaging of ∼1 mm deep into the biological tissue. Selection criteria of the coating include high transparency and low scattering (Section 1 and Section 3.3), diffusion of analyte into the coating (Section 1), biocompatibility (see Section 4.2)
How (‘future solutions’)	3D printed micro-optics/lenses	Creating complex shape, which has optimized lens design for sensing and imaging, directly on the tip of a fiber with fast and reliable transfer from design to production
	Specialty microstructured optical fibers (MOF)	Taking advantage of the high sensitivity of MOFs while supporting single-mode OCT imaging
	Coating with low attenuation	Spatial confinement of the coating to the sensing region to minimize image distortions
	Photoswitchable coatings	For image-guided sensing in dynamic in vivo applications: the sensing function is only switched-on when the imaging probe has identified the right location for sensing
	2D materials	High surface area-to-volume ratios and adaptable optical properties to enhance the sensitivity and imaging performance of single-fiber-based sensing + imaging probes
	Combination of other modalities	Multimode fiber imaging, fluorescence lifetime-based imaging, photoacoustic tomography/sensing, and Raman scattering based sensing

## Data Availability

Data may be obtained from the authors upon reasonable request.

## Supplemental document

See Supplement 1 for supporting content.

## References

[1] Min R. Liu Z. Pereira L. et al., “Optical fiber sensing for marine environment and marine structural health monitoring: A review,” Opt. Laser Technol. 140, 107082 (2021). 10.1016/j.optlastec.2021.107082

[2] Correia R. James S. Lee S. W. et al., “Biomedical application of optical fibre sensors,” J. Opt. 20(7), 073003 (2018). 10.1088/2040-8986/aac68d

[3] Mazzarello P., “A unifying concept: the history of cell theory,” Nat. Cell Biol. 1(1), E13–E15 (1999). 10.1038/8964 10559875

[4] Stosiek C. Garaschuk O. Holthoff K. et al., “In vivo two-photon calcium imaging of neuronal networks,” Proc. Natl. Acad. Sci. 100(12), 7319–7324 (2003). 10.1073/pnas.1232232100 12777621 PMC165873

[5] Beaudette K. Li J. Lamarre J. et al., “Double-clad fiber-based multifunctional biosensors and multimodal bioimaging systems: technology and applications,” Biosensors 12(2), 90 (2022). 10.3390/bios12020090 35200350 PMC8869713

[6] Li J. Ebendorff-Heidepriem H. Gibson B. C. et al., “Perspective: Biomedical sensing and imaging with optical fibers—Innovation through convergence of science disciplines,” APL Photonics 3(10), 100902 (2018). 10.1063/1.5040861

[7] Li J. Schartner E. Musolino S. et al., “Miniaturized single-fiber-based needle probe for combined imaging and sensing in deep tissue,” Opt. Lett. 43(8), 1682–1685 (2018). 10.1364/OL.43.001682 29652339

[8] Bronk K. S. Michael K. L. Pantano P. et al., “Combined imaging and chemical sensing using a single optical imaging fiber,” Anal. Chem. 67(17), 2750–2757 (1995). 10.1021/ac00113a005 8779411

[9] Michael K. L. Taylor L. C. Walt D. R., “A far-field-viewing sensor for making analytical measurements in remote locations,” Anal. Chem. 71(14), 2766–2773 (1999). 10.1021/ac990085q 10424167

[10] Michael K. L. Walt D. R., “Combined imaging and chemical sensing of fertilization-induced acid release from single sea urchin eggs,” Anal. Biochem. 273(2), 168–178 (1999). 10.1006/abio.1999.4173 10469487

[11] Issberner J. P. Schauer C. L. Trimmer B. A. et al., “Combined imaging and chemical sensing of l-glutamate release from the foregut plexus of the lepidopteran, manduca sexta,” J. Neurosci. Methods 120(1), 1–10 (2002). 10.1016/S0165-0270(02)00165-6 12351201

[12] Drexler W. Fujimoto J. G., *Optical Coherence Tomography: Technology and Applications* (Springer Science & Business Media, 2015).

[13] Capon P. K. Li J. Horsfall A. J. et al., “A Silk-Based Functionalization Architecture for Single Fiber Imaging and Sensing,” Adv. Funct. Mater. 32(3), 2010713 (2022). 10.1002/adfm.202010713

[14] Khalid A. Peng L. Arman A. et al., “Silk: A bio-derived coating for optical fiber sensing applications,” Sens. Actuators, B 311, 127864 (2020). 10.1016/j.snb.2020.127864

[15] Yoo H. Kim J. W. Shishkov M. et al., “Intra-arterial catheter for simultaneous microstructural and molecular imaging in vivo,” Nat. Med. 17(12), 1680–1684 (2011). 10.1038/nm.2555 22057345 PMC3233646

[16] Ueno T. Nagano T., “Fluorescent probes for sensing and imaging,” Nat. Methods 8(8), 642–645 (2011). 10.1038/nmeth.1663 21799499

[17] François A. Ebendorff-Heidepriem H. Monro T. M., *Comparison of Surface Functionalization Processes for Optical Fibre Biosensing Applications* (SPIE 2009), pp. 128–131.

[18] Ruan Y. Foo T. C. Warren-Smith S. et al., “Antibody immobilization within glass microstructured fibers: a route to sensitive and selective biosensors,” Opt. Express 16(22), 18514–18523 (2008). 10.1364/OE.16.018514 18958130

[19] Kostecki R. Ebendorff-Heidepriem H. Shahraam et al., “Novel polymer functionalization method for exposed-core optical fiber,” Opt. Mater. Express 4(8), 1515–1525 (2014). 10.1364/OME.4.001515

[20] Chen M. Wang J. Tan W. et al., “Miniaturized all fiber probe for optical coherence tomography and pH detection of biological tissue,” J. Biophotonics 14(2), e202000239 (2021). 10.1002/jbio.202000239 33048463

[21] Jakutis J. Gomes L. Amancio C. et al., “Increased Er^3+^ upconversion in tellurite fibers and glasses by co-doping with Yb^3+^,” Opt. Mater. 33(1), 107–111 (2010). 10.1016/j.optmat.2010.08.021

[22] Kang J. U. Han J. H. Liu X. et al., “Endoscopic Functional Fourier Domain Common-Path Optical Coherence Tomography for Microsurgery,” IEEE J. Sel. Top. Quantum Electron. 16(4), 781–792 (2010). 10.1109/JSTQE.2009.2031597 22899880 PMC3418670

[23] Ruan Y. Simpson D. A. Jeske J. et al., “Magnetically sensitive nanodiamond-doped tellurite glass fibers,” Sci. Rep. 8(1), 1268 (2018). 10.1038/s41598-018-19400-3 29352215 PMC5775195

[24] Purdey M. S. Thompson J. G. Monro T. M. et al., “A dual sensor for pH and hydrogen peroxide using polymer-coated optical fibre tips,” Sensors 15(12), 31904–31913 (2015). 10.3390/s151229893 26694413 PMC4721812

[25] Schartner E. P. Henderson M. R. Purdey M. et al., “Cancer Detection in Human Tissue Samples Using a Fiber-Tip pH Probe,” Cancer Res. 76(23), 6795–6801 (2016). 10.1158/0008-5472.CAN-16-1285 27903493

[26] Kostecki R. Heng S. Mak A. M. et al., “Control of Molecular Recognition via Modulation of the Nanoenvironment,” ACS Appl. Mater. Interfaces 10(49), 41866–41870 (2018). 10.1021/acsami.8b16161 30431255

[27] Capon P. K. Horsfall A. J. Li J. et al., “Protein detection enabled using functionalised silk-binding peptides on a silk-coated optical fibre,” RSC Adv. 11(36), 22334–22342 (2021). 10.1039/D1RA03584C 35480827 PMC9034238

[28] Scolaro L. Lorenser D. Madore W.-J. et al., “Molecular imaging needles: dual-modality optical coherence tomography and fluorescence imaging of labeled antibodies deep in tissue,” Biomed. Opt. Express 6(5), 1767–1781 (2015). 10.1364/BOE.6.001767 26137379 PMC4467702

[29] Li J. Thiele S. Kirk R. W. et al., “3D-Printed Micro Lens-in-Lens for In Vivo Multimodal Microendoscopy,” Small 18(17), 2107032 (2022). 10.1002/smll.202107032 35229467

[30] Warren-Smith S. C. Afshar S. Monro T. M., “Fluorescence-based sensing with optical nanowires: a generalized model and experimental validation,” Opt. Express 18(9), 9474–9485 (2010). 10.1364/OE.18.009474 20588793

[31] Shahraam Afshar V. Warren-Smith S. C. Monro T. M., “Enhancement of fluorescence-based sensing using microstructured optical fibres,” Opt. Express 15(26), 17891–17901 (2007). 10.1364/OE.15.017891 19551084

[32] Zhao J. Jin D. Schartner E. P. et al., “Single-nanocrystal sensitivity achieved by enhanced upconversion luminescence,” Nat. Nanotechnol. 8(10), 729–734 (2013). 10.1038/nnano.2013.171 23995455

[33] Wang J. Yang X. Wang L., “Fabrication and experimental observation of monolithic multi-air-core fiber array for image transmission,” Opt. Express 16(11), 7703–7708 (2008). 10.1364/OE.16.007703 18545479

[34] Warren-Smith S. C. Dowler A. Ebendorff-Heidepriem H., “Soft-glass imaging microstructured optical fibers,” Opt. Express 26(26), 33604–33612 (2018). 10.1364/OE.26.033604 30650793

[35] Wood H. A. C. Harrington K. Birks T. A. et al., “High-resolution air-clad imaging fibers,” Opt. Lett. 43(21), 5311–5314 (2018). 10.1364/OL.43.005311 30383002

[36] Schartner E. P. Tsiminis G. Henderson M. R. et al., “Quantification of the fluorescence sensing performance of microstructured optical fibers compared to multi-mode fiber tips,” Opt. Express 24(16), 18541–18550 (2016). 10.1364/OE.24.018541 27505817

[37] Warren-Smith S. Heng S. Ebendorff-Heidepriem H. et al., “Fluorescence-based aluminum ion sensing using a surface-functionalized microstructured optical fiber,” Langmuir 27(9), 5680–5685 (2011). 10.1021/la2002496 21469740

[38] Heng S. McDevitt C. A. Kostecki R. et al., “Microstructured optical fiber-based biosensors: reversible and nanoliter-scale measurement of zinc ions,” ACS Appl. Mater. Interfaces 8(20), 12727–12732 (2016). 10.1021/acsami.6b03565 27152578

[39] Heng S. Mak A. M. Stubing D. B. et al., “Dual Sensor for Cd(II) and Ca(II): Selective Nanoliter-Scale Sensing of Metal Ions,” Anal. Chem. 86(7), 3268–3272 (2014). 10.1021/ac500619z 24617734

[40] Wong W. S. Peng X. McLaughlin J. M. et al., “Breaking the limit of maximum effective area for robust single-mode propagation in optical fibers,” Opt. Lett. 30(21), 2855–2857 (2005). 10.1364/OL.30.002855 16279448

[41] Warren-Smith S. C. Ebendorff-Heidepriem H. Foo T. C. et al., “Exposed-core microstructured optical fibers for real-time fluorescence sensing,” Opt. Express 17(21), 18533–18542 (2009). 10.1364/OE.17.018533 20372584

[42] Richardson D. J. Nilsson J. Clarkson W. A., “High power fiber lasers: current status and future perspectives [Invited],” J. Opt. Soc. Am. B 27(11), B63–B92 (2010). 10.1364/JOSAB.27.000B63

[43] Limpert J. Schreiber T. Nolte S. et al., “High-power air-clad large-mode-area photonic crystal fiber laser,” Opt. Express 11(7), 818–823 (2003). 10.1364/OE.11.000818 19461794

[44] Sahu J. K. Renaud C. C. Furusawa K. et al., “Jacketed air-clad cladding pumped ytterbium-doped fibre laser with wide tuning range,” in *Electronics Letters* (Institution of Engineering and Technology, 2001), pp. 1116–1117.

[45] Furusawa K. Malinowski A. Price J. H. V. et al., “Cladding pumped Ytterbium-doped fiber laser with holey inner and outer cladding,” Opt. Express 9(13), 714–720 (2001). 10.1364/OE.9.000714 19424311

[46] Dong H. Gao W. Yan F. et al., “Fluorescence resonance energy transfer between quantum dots and graphene oxide for sensing biomolecules,” Anal. Chem. 82(13), 5511–5517 (2010). 10.1021/ac100852z 20524633

[47] Stich M. I. J. Fischer L. H. Wolfbeis O. S., “Multiple fluorescent chemical sensing and imaging,” Chem. Soc. Rev. 39(8), 3102–3114 (2010). 10.1039/b909635n 20571676

[48] Huang Y. Cao X. Deng Y. et al., “An overview on recent advances of reversible fluorescent probes and their biological applications,” Talanta 268, 125275 (2024). 10.1016/j.talanta.2023.125275 37839322

[49] Heng S. Mak A. M. Kostecki R. et al., “Photoswitchable calcium sensor: ‘On’–‘Off’ sensing in cells or with microstructured optical fibers,” Sens. Actuators, B 252, 965–972 (2017). 10.1016/j.snb.2017.06.051

[50] Sylvia G. M. Mak A. M. Heng S. et al., “A rationally designed, spiropyran-based chemosensor for magnesium,” Chemosensors 6(2), 17 (2018). 10.3390/chemosensors6020017

[51] Anichini C. Czepa W. Pakulski D. et al., “Chemical sensing with 2D materials,” Chem. Soc. Rev. 47(13), 4860–4908 (2018). 10.1039/C8CS00417J 29938255

[52] Ares P. Novoselov K. S., “Recent advances in graphene and other 2D materials,” Nano Mater. Sci. 4(1), 3–9 (2022). 10.1016/j.nanoms.2021.05.002

[53] Qian Z. S. Shan X. Y. Chai L. J. et al., “A fluorescent nanosensor based on graphene quantum dots-aptamer probe and graphene oxide platform for detection of lead (II) ion,” Biosens. Bioelectron. 68, 225–231 (2015). 10.1016/j.bios.2014.12.057 25574861

[54] Wen Y. Xing F. He S. et al., “A graphene-based fluorescent nanoprobe for silver(I) ions detection by using graphene oxide and a silver-specific oligonucleotide,” Chem. Commun. 46(15), 2596–2598 (2010). 10.1039/b924832c 20449319

[55] Liu X. Li L. Wei Y. et al., “Facile synthesis of boron- and nitride-doped MoS_2_ nanosheets as fluorescent probes for the ultrafast, sensitive, and label-free detection of Hg(2+),” Analyst 140(13), 4654–4661 (2015). 10.1039/C5AN00641D 25988202

[56] Singh R. Zhang W. Liu X. et al., “Humanoid-shaped WaveFlex biosensor for the detection of food contamination,” Biomed. Opt. Express 14(9), 4660–4676 (2023). 10.1364/BOE.500311 37791266 PMC10545203

[57] Lu C. H. Yang H. H. Zhu C. L. et al., “A graphene platform for sensing biomolecules,” Angew. Chem., Int. Ed. 48(26), 4785–4787 (2009). 10.1002/anie.200901479 19475600

[58] Zhang Y. Lin H. Meng F. et al., “An ultrahigh sensitivity micro-cliff graphene wearable pressure sensor made by instant flash light exposure,” Nanoscale 13(36), 15380–15393 (2021). 10.1039/D1NR04333A 34499073

[59] Han J. Lin K.-T. Lin H. et al., “Tunable thermochromic graphene metamaterials with iridescent color,” Nano Lett. 22(14), 6026–6033 (2022). 10.1021/acs.nanolett.1c04768 35639615

[60] Cao G., *On Fibre Tip Graphene-based Ultrathin Flat Lens Towards High-Resolution Endoscopes* (Swinburne University of Technology, 2020).

[61] Du B. Ruan Y. Ly T.-T. et al., “MoS2-enhanced epoxy-based plasmonic fiber-optic sensor for selective and sensitive detection of methanol,” Sens. Actuators, B 305, 127513 (2020). 10.1016/j.snb.2019.127513

[62] Chen J.-H. Xiong Y.-F. Xu F. et al., “Silica optical fiber integrated with two-dimensional materials: towards opto-electro-mechanical technology,” Light: Sci. Appl. 10(1), 78 (2021). 10.1038/s41377-021-00520-x 33854031 PMC8046821

[63] Plöschner M. Tyc T. Čižmár T., “Seeing through chaos in multimode fibres,” Nat. Photonics 9(8), 529–535 (2015). 10.1038/nphoton.2015.112

[64] Wen Z. Dong Z. Deng Q. et al., “Single multimode fibre for in vivo light-field-encoded endoscopic imaging,” Nat. Photonics 17(8), 679–687 (2023). 10.1038/s41566-023-01240-x

[65] Bec J. Phipps J. E. Gorpas D. et al., “In vivo label-free structural and biochemical imaging of coronary arteries using an integrated ultrasound and multispectral fluorescence lifetime catheter system,” Sci. Rep. 7(1), 8960 (2017). 10.1038/s41598-017-08056-0 28827758 PMC5566546

[66] Wang L. V. Hu S., “Photoacoustic tomography: in vivo imaging from organelles to organs,” Science 335(6075), 1458–1462 (2012). 10.1126/science.1216210 22442475 PMC3322413

[67] Zhou J. Jokerst J. V., “Photoacoustic imaging with fiber optic technology: A review,” Photoacoustics 20, 100211 (2020). 10.1016/j.pacs.2020.100211 33163358 PMC7606844

[68] Duan T. Lan H. Zhong H. et al., “Hybrid multi-wavelength nonlinear photoacoustic sensing and imaging,” Opt. Lett. 43(22), 5611–5614 (2018). 10.1364/OL.43.005611 30439907

[69] Fitzgerald S. Akhtar J. Schartner E. et al., “Multimodal Raman spectroscopy and optical coherence tomography for biomedical analysis,” J. Biophotonics 16(3), e202200231 (2023). 10.1002/jbio.202200231 36308009 PMC10082563

[70] Ehrlich K. Kufcsák A. McAughtrie S. et al., “pH sensing through a single optical fibre using SERS and CMOS SPAD line arrays,” Opt. Express 25(25), 30976–30986 (2017). 10.1364/OE.25.030976 29245776

[71] Bourantas C. V. Jaffer F. A. Gijsen F. J. et al., “Hybrid intravascular imaging: recent advances, technical considerations, and current applications in the study of plaque pathophysiology,” Eur. Heart J. 38(6), 400–412 (2017). 10.1093/eurheartj/ehw097 27118197 PMC5837589

[72] Li J. Montarello N. J. Hoogendoorn A. et al., “Multimodality intravascular imaging of high-risk coronary plaque,” JACC: Cardiovascular Imaging 15(1), 145–159 (2022). 10.1016/j.jcmg.2021.03.028 34023267

[73] Tian K. Zhang M. Zhao Z. et al., “Ultra-compact in-core-parallel-written FBG and Mach–Zehnder interferometer for simultaneous measurement of strain and temperature,” Opt. Lett. 46(22), 5595–5598 (2021). 10.1364/OL.440118 34780414

[74] Nazıroğlu M. Dikici D. M. Dursun Ş., “Role of oxidative stress and Ca^2 ^ signaling on molecular pathways of neuropathic pain in diabetes: focus on TRP channels,” Neurochem. Res. 37(10), 2065–2075 (2012). 10.1007/s11064-012-0850-x 22846968

[75] Resnick L. M., “Cellular ions in hypertension, insulin resistance, obesity, and diabetes: a unifying theme,” J. Am. Soc. Nephrol. 3(4), S78 (1992). 10.1681/ASN.V34s78 1457764

[76] Nozaki C. Vergnano A. M. Filliol D. et al., “Zinc alleviates pain through high-affinity binding to the NMDA receptor NR2A subunit,” Nat. Neurosci. 14(8), 1017–1022 (2011). 10.1038/nn.2844 21725314 PMC4494785

[77] Li J. Quirk B. C. Noble P. B. et al., “Flexible needle with integrated optical coherence tomography probe for imaging during transbronchial tissue aspiration,” J. Biomed. Opt. 22(10), 106002 (2017). 10.1117/1.JBO.22.10.106002 29022301

[78] Curley S. A. Izzo F. Delrio P. et al., “Radiofrequency ablation of unresectable primary and metastatic hepatic malignancies: results in 123 patients,” Ann. Surg. 230(1), 1–8 (1999). 10.1097/00000658-199907000-00001 10400029 PMC1420837

[79] Dewhirst M. W. Viglianti B. L. Lora-Michiels M. et al., “Thermal dose requirement for tissue effect: experimental and clinical findings,” Proceedings of SPIE–the International Society for Optical Engineering 4954, 37 (2003). 10.1117/12.476637 25301982 PMC4188373

[80] Ahmed M. Brace C. L. Lee Jr. F. T. et al., “Principles of and advances in percutaneous ablation,” Radiology 258(2), 351–369 (2011). 10.1148/radiol.10081634 21273519 PMC6939957

[81] Ramakonar H. Quirk B. C. Kirk R. W. et al., “Intraoperative detection of blood vessels with an imaging needle during neurosurgery in humans,” Sci. Adv. 4(12), eaav4992 (2018). 10.1126/sciadv.aav4992 30585293 PMC6300404

[82] Walther J. Golde J. Albrecht M. et al., “A handheld fiber-optic probe to enable optical coherence tomography of oral soft tissue,” IEEE Trans. Biomed. Eng. 69(7), 2276–2282 (2022). 10.1109/TBME.2022.3141241 34995178

[83] Scolaro L. Lorenser D. Quirk B. C. et al., “Multimodal imaging needle combining optical coherence tomography and fluorescence for imaging of live breast cancer cells labeled with a fluorescent analog of tamoxifen,” J. Biomed. Opt. 27(07), 076004 (2022). 10.1117/1.JBO.27.7.076004 35831923 PMC9278982

[84] Vidanapathirana A. K. Goyne J. M. Williamson A. E. et al., “Biological Sensing of Nitric Oxide in Macrophages and Atherosclerosis Using a Ruthenium-Based Sensor,” Biomedicines 10(8), 1807 (2022). 10.3390/biomedicines10081807 36009353 PMC9405170

[85] Mackle E. C. Coote J. M. Carr E. et al., “Fibre optic intravascular measurements of blood flow: A review,” Sens. Actuators, A 332, 113162 (2021). 10.1016/j.sna.2021.113162

[86] Nazempour R. Zhang Q. Fu R. et al., “Biocompatible and Implantable Optical Fibers and Waveguides for Biomedicine,” Materials 11(8), 1283 (2018). 10.3390/ma11081283 30044416 PMC6117721

[87] Jian M. Zhang Y. Liu Z., “Natural Biopolymers for Flexible Sensing and Energy Devices,” Chin. J. Polym. Sci. 38(5), 459–490 (2020). 10.1007/s10118-020-2379-9

[88] Nguyen T. T. Zhang P. Bi J. et al., “Silver– Gallium Nano-Amalgamated Particles as a Novel, Biocompatible Solution for Antibacterial Coatings,” Adv. Funct. Mater. 2310539, 1 (2023). 10.1002/adfm.202310539

[89] Pham T. Nguyen T. T. Nguyen N. H. et al., “Transforming Spirulina maxima Biomass into Ultrathin Bioactive Coatings Using an Atmospheric Plasma Jet: A New Approach to Healing of Infected Wounds,” Small 2305469, 1 (2023). 10.1002/smll.202305469 37715087

