# Supplementary material for: Single-fiber probes for combined sensing and imaging in biological tissue: recent developments and prospects
Source: Biomed Opt Express. 2024 Mar 15;15(4):2392–405. doi: 10.1364/BOE.517920 (PMC11019705; doi:10.1364/BOE.517920)
Supplement: Supplementary file 1 [file boe-15-4-2392-s001.pdf]

Supplemental Document

Biomedical Optics EXPRESS

Single-fiber probes for combined sensing and imaging in biological tissue: recent developments and prospects: supplement

JIAWEN LI,[1,2,*] STEPHEN C. WARREN-SMITH,[2,3] ROBERT A. MCLAUGHLIN,[2,4] AND HEIKE EBENDORFF-HEIDEPRIEM[2,5]

[1] *School of Electrical and Mechanical Engineering, The University of Adelaide, South Australia, 5005, Australia*
[2] *Institute for Photonics and Advanced Sensing, The University of Adelaide, South Australia, 5005, Australia*
[3] *Future Industries Institute, The University of South Australia, Mawson Lakes, South Australia, 5095, Australia*
[4] *Faculty of Health and Medical Sciences, The University of Adelaide, South Australia, 5005, Australia*
[5] *School of Physics, Chemistry and Earth Sciences, The University of Adelaide, South Australia, 5005, Australia*
[*] *jiawen.li01@adelaide.edu.au*

This supplement published with Optica Publishing Group on 15 March 2024 by The Authors under the terms of the Creative Commons Attribution 4.0 License in the format provided by the authors and unedited. Further distribution of this work must maintain attribution to the author(s) and the published article's title, journal citation, and DOI.

Supplement DOI: https://doi.org/10.6084/m9.figshare.25310965

Parent Article DOI: https://doi.org/10.1364/BOE.517920

Single-fiber probes for combined sensing and imaging in biological tissue: recent developments and prospects

JIAWEN LI,[1,2,*] STEPHEN WARREN-SMITH,[3] ROBERT A. MCLAUGHLIN,[2,4] AND HEIKE EBENDORFF-HEIDEPRIEM[2,5]

[1]*School of Electrical and Mechanical Engineering and Institute for Photonics and Advanced Sensing, The University of Adelaide, South Australia, 5005, Australia*
[2]*Australian Research Council Centre of Excellence for Nanoscale BioPhotonics, Australia*
[3]*Future Industries Institute at the University of South Australia, Mawson Lakes, South Australia, 5095, Australia*
[4]*Adelaide Medical School, Faculty of Health and Medical Sciences, The University of Adelaide, South Australia, 5005, Australia*
[5]*School of Physics, Chemistry and Earth Sciences and Institute for Photonics and Advanced Sensing, The University of Adelaide, South Australia, 5005, Australia*
**jiawen.li01@adelaide.edu.au*

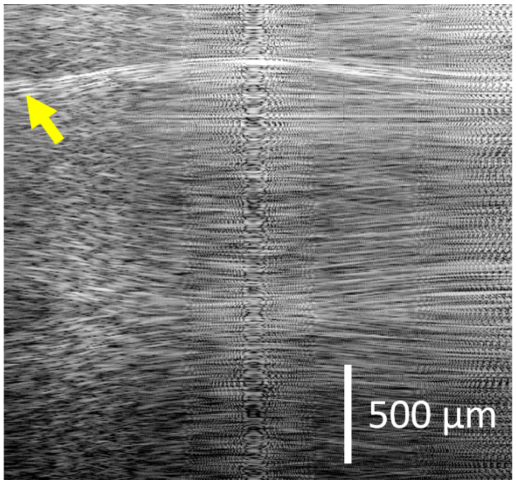

Fig. S1. OCT image obtained with a polyacrylamide coating on the end of a double clad fiber, which can be used to collect both fluorescence-based sensing and imaging signals (if any). Yellow arrow points at the sample (IR card) being imaged, but the signal of this sample was buried in strong noise caused by the coating.
